# Trastuzumab Mediated T-Cell Response against HER-2/Neu Overexpressing Esophageal Adenocarcinoma Depends on Intact Antigen Processing Machinery

**DOI:** 10.1371/journal.pone.0012424

**Published:** 2010-08-26

**Authors:** Francesca Milano, Mirta Guarriera, Agnieszka M. Rygiel, Kausilia K. Krishnadath

**Affiliations:** 1 Center of Experimental and Molecular Medicine, Academic Medical Center, Amsterdam, The Netherlands; 2 Department of Gastroenterology and Hepatology, Academic Medical Center, Amsterdam, The Netherlands; Centre de Recherche Public de la Santé, Luxembourg

## Abstract

**Background:**

Esophageal adenocarcinoma (EAC) is a highly aggressive disease with poor prognosis, which frequently exhibits HER-2 gene amplification. Trastuzumab, the humanized antibody against HER-2, has potent growth inhibitory effects on HER-2 overexpressing cancers. One effect of trastuzumab is that it causes HER-2 receptor internalization and degradation, enhancing presentation of HER-2 epitopes on MHC-Class I molecules. This enhances the ability of HER-2 specific cytotoxic T lymphocytes (CTLs) to recognize and kill cancer cells. Novel strategies targeting the HER-2 receptor either directly by trastuzumab and/or indirectly by inducing a CTL response against HER-2 epitopes with, for instance, DC immunotherapy and consequently combining these strategies might prove to be very effective.

**Methodology/Principal Findings:**

In this study we report that trastuzumab has potent growth inhibitory effects on two HER-2 overexpressing EAC cell lines OE33 and OE19. However, we found that trastuzumab and HER-2 specific CTLs act synergistically in inducing tumor lysis in OE33 but not in OE19. We discovered that in OE19 this deficient response is due to a down-regulation of the Transporter Associated with Antigen Processing-2 (TAP-2). TAP-2 is an important member of the Antigen Processing Machinery (APM), and is one of the essential elements for loading antigens on MHC class I molecules. Importantly, we demonstrated that by inducing re-expression of TAP-2 in OE19 with INF-γ treatment or by incubating the cells with INF-γ producing CTLs, the specific anti HER-2 CTL tumor lysis response and synergistic effect with trastuzumab can be restored.

**Conclusion:**

An inefficient response of HER-2 overexpressing EAC to trastuzumab and/or DC immunotherapy can be due to a down-regulated TAP-2 expression and thus a deficient APM. Future studies combining trastuzumab with IFN-γ and/or immune-therapies inducing potent anti HER-2 CTL responses could lead to an effective combinatorial strategy for successful treatment of HER-2 overexpressing but APM defective cancers.

## Introduction

HER-2/neu is a 185 KDa transmembrane glycoprotein with tyrosine kinase activity[1]. It is overexpressed, mostly via gene amplification, in several aggressive cancers [2], such as in 25–30% of ovarian and breast cancers [3], [4], 35–45% of pancreatic carcinomas [5], and in 30–80% of EAC [6]–[9]. Interestingly, cytotoxic T-lymphocyte (CTL) responses against several HER-2 peptides have been observed in cancer patients, indicating that the HER-2/neu protein is immunogenic. Therefore, the HER-2/neu receptor is regarded as an ideal Tumor Associated Antigen, which might be employed for anti-cancer immunotherapy [10], [11], [12]. Moreover, targeting of the HER-2 receptor by the humanized antibody trastuzumab has been shown to result in potent growth inhibition of HER-2 overexpressing tumors [13], [14]. In clinical studies trastuzumab has been shown to give significant results, particularly in the treatment of breast cancer [15], [16]. Applying trastuzumab as an additional treatment option is an attractive strategy for tumors with poor prognosis bearing HER-2 overexpression, such as EAC. In Western countries, this cancer has the most rapidly increasing incidence compared to other cancers [17], [18]. Currently, the main treatment for this disease is surgical resection, yet, even after surgery with or without neo-adjuvant or adjuvant chemo- and radiotherapy, the median survival of these patients is less than 2 years [19], [20]. Recently, several studies suggest that therapies targeting HER-2 either by specific anti-HER-2 antibodies or induced anti-HER-2 CTLs or a combination of these treatments may be effective (neo) adjuvant treatments for HER-2 positive cancers [13], [14]. At present, only a few reports have explored the possibility of applying trastuzumab as an additional treatment option for EAC patients in phase I/II studies. Results from this study are still pending, however in the initial phase the treatment induced an overall toxicity, which was not increased as compared to previous studies using this antibody [21].

The underlying mechanisms of action of trastuzumab are diverse and not yet fully understood. One recognized effect of trastuzumab is the enhancement of the immune system response such as Antibody Dependent Cellular Cytotoxicity (ADCC) [22], [23]. Yet, another lesser known, but crucial effect of trastuzumab is the enhancement of the MHC-Class I restricted HER-2 epitope presentation on tumor cells. This results in a boosted HER-2 specific CTL response and, consequently, increased tumor cell lysis. In this regard, it has been previously shown that HER-2 overexpressing gastric cell lines and esophageal squamous cancer cells treated with trastuzumab are sensitized and more susceptible to killing by HER-2 specific CTLs [24], [25].

We previously demonstrated in an ex-vivo model that DC mediated CTL responses could be an advantageous approach for improving EAC treatment [26]. Both DC immunotherapy and the use of trastuzumab in the clinic has resulted so far in partly improved patient responses, however, these were undeniably below expectations.

In this study, we questioned whether DC mediated anti HER-2 specific CTL immunotherapy combined with trastuzumab could be an even more effective strategy for treatment of EAC. To this aim, we firstly examined the level of gene amplification and protein overexpression in EAC cell lines by Fluorescence In Situ Hybridization (FISH) and immunocytochemistry (ICC). We then evaluated the effect of trastuzumab on HER-2 overexpressing EAC cell lines. Furthermore, we studied whether trastuzumab sensitizes HER-2 overexpressing tumor cells to lysis by HER-2 specific CTLs. Remarkably, in one of the HER-2 positive EAC cell lines OE33 we observed a synergy between trastuzumab treatment and specific anti-HER-2 CTL responses. However, the other EAC cell line OE19, was not sensitive to HER-2 specific CTLs also without trastuzumab pre-treatment. Investigation of the status of several components of the Antigen Processing Machinery (APM) in this cell line and in EAC tumor biopsy specimens showed that the expression of one of the most important components of the APM, the Transporter Associated with Antigen Processing-2 (TAP-2) is specifically down regulated in OE19 and also in a high percentage of EAC. Importantly, we show that TAP-2 can be re-induced not only by IFN-γ treatment, but also by potent IFN-γ producing CTL populations. Importantly, up-regulation of TAP-2 restored the synergistic cytotoxic effect of trastuzumab and the anti HER-2 CTL response in OE19. We further show that the HER-2 specific CTLs induced cytotoxicity, in synergy with trastuzumab, specifically depends on an intact APM, in view of the fact that down-regulation of TAP-2 expression in the TAP-2 proficient cell line OE33 hinders the synergistic cytotoxic effect induced by CTLs and trastuzumab. In the future, it will be of importance to assess the levels of TAP-2 expression in EAC patient in order to improve clinical responses to DC immunotherapy and trastuzumab or combinatorial targeted therapies.

## Materials and Methods

### Cell Lines

EAC cell lines OE19 and OE33 were obtained from ECACC (Porton Down, Wiltshire, SP4 DJG, UK), SW620, a colorectal adenocarcinoma cell line used as control [27], was a kind gift from Dr. David Beer (University of Washington, USA). OE19 and OE33 were maintained in RPMI 1640 medium with addition of L-glutamine, antibiotics and Fetal Calf Serum (FCS). SW620 was kept in DMEM medium with addition of L-glutamine, antibiotics and FCS. All the cell lines were cultured at 37°C at 5% CO_2_ and medium was refreshed every three days. The HLA-A2 status of the cell lines was determined by FACS analysis using an anti-human HLA-A2 antibody FITC conjugated (BD, The Netherlands). The FACS Staining procedure was performed as described previously [26].

### Biopsy specimens from EAC patients

The study was approved by the Academic Medical Center Hospital's Medical Ethical Committee, Amsterdam, The Netherlands. After informed consent and written permission, sixteen patients were included. Patients underwent endoscopic procedures for classifying, staging and grading of the esophageal cancer. During this procedure, 3 extra biopsies of each patient were taken to be used for experimental purposes; biopsies were obtained from both normal squamous epithelium taken at least 3 cm above the malignant mass, and from the malignancy itself. Matching biopsies from the same spots were taken for histopathological diagnosis. Biopsies were collected in Trizol and RNA was extracted as previously described [26].

### Fluorescent *in situ* hybridization (FISH)

Upon reaching a confluence of 70–80%, EAC cell lines OE33, OE19 and SW620 were trypsinized, spun down and collected in PBS. A Cytospin (Shandon Cytospin 4 Cytocentrifuge, Thermo, Waltham, MA) was used to generate a single layer of cells on a glass slide as described before (45). We used directly labeled fluorescent chromosomal centromeric probes (CEP) for chromosome 17 and 17q11.2- q12 (*Her-2/neu*), obtained from Vysis (Downers Grove, IL). DNA-FISH was performed according to the manufacturer's instructions provided by Vysis as described before by our group [28].

### Fluorescent immuno-cytochemistry

Cultured EAC cells were plated in a 24 well plate at a concentration of 2, 5×10^4^/ml and grown on coverslips. After 24 hours, cultured cells were fixed directly in the well for 20 minutes in Phosphate Buffered Saline (PBS) with 4% Paraformaldehyde (PFA) and 0.1% Triton X-100 and then washed in PBS. Blocking of aspecific antigens was performed by incubating slides for 45 minutes with PBS containing 1% BSA and 10% Fetal Calf Serum (FCS). Slides were washed with PBS and incubated overnight at 4°C with the appropriately diluted primary HER-2 antibody, c-erbB-2/HER-2/neuAb-17 (Lab Vision Corporation, Freemont, CA, USA) in PBS with 1% BSA and 0.1% Triton X-100. After washing with PBS, cells were incubated with a mouse anti-human secondary FITC conjugated antibody (Dako, Denmark) diluted 1∶500 in PBS. Cells attached on coverslips were placed on Superfrost + (Menzel Glaser, Braunschweig, Germany) glass slides and mounted with DAPI (Roche, Mannheim, Germany)/vectashield (Vector laboratories Inc, Burlingame, CA, USA).

### Generation of HER-2 mRNA by *in vitro* transcription (IVT)

HER-2 mRNA was made as previously described [29]. Briefly, to generate HER-2 mRNA, plasmid pSPJC1 with an insert coding for HER-2 (a gift from Prof. A. Ulrich, Martinsried, Germany) was used. This plasmid allows in vitro transcription under the control of an SP6 promoter. The plasmid was linearized using NdeI (MBI Fermentas, St. Leon-Rot, Germany) and in vitro transcription was subsequently performed using Ambion in vitro transcription kit with an SP6 promoter (Ambion, Nieuwerkerk a/d, IJssel, The Netherlands), and the manufacturer's procedure was followed. The in vitro transcribed mRNA was purified using RNeasy Midi Kit (Qiagen, Hilden, Germany) according to the manufacture's instructions. The RNA was then dissolved in RNase-free H_2_O and stored at −80°C until further use. Total RNA was quantified using the Nanodrop® according to the manufacturer's instructions. (Nanodrop Tecnologies, type ND-1000, Wilmington, USA).

### Maturation of HER-2 mRNA loaded Dendritic Cells and Induction of anti HER-2 specific CTLs

HLA-A2 matching monocytes obtained from a buffy coat and characterized as CD14+, CD83−, CD86−, CD209− were used for electroporation with HER-2 mRNA. The electroporation procedure and the isolation of monocytes and maturation of DCs were performed as previously described [30]. The electroporation efficacy of HER-2 mRNA was assessed through measuring HER-2 expression by FACS analysis. FACS staining was performed as described before [26]. A mouse anti-human HER-2 primary monoclonal antibody, clone 9G6.10 (Lab Vision, Fremont, CA), an anti-mouse PE conjugated antibody, and an isotype control for the anti-mouse PE secondary antibody were used. Expression was measured in monocytes 24 hours after electroporation (day one) and eight days after electroporation.

The obtained HER-2 loaded mature DCs were twice co-incubated for seven days with autologous lymphocytes in a ratio of 1∶4 (5×10^5^ DCs and 2×10^6^ lymphocytes) in 24 well plates in AIM V medium with 2% penicillin-streptomycin (GIBCO) without FCS. To obtain a control population, lymphocytes were also incubated with mock transfected DCs.

### Mixed Leucocyte Reaction (MLR) and [^3^H] thymidine incorporation assay

Mature DCs electroporated with HER-2 RNA and mock DCs were co-cultured in flat-bottom 96-well plates (Nunc, Roskilde, Denmark) with lymphocytes in IMDM medium at different target-effector ratios, starting from 4×10^3^/well, down to 1.25×10^2^ DCs per well, while each well contained a fixed number of 5×10^4^ lymphocytes. After 5 days in culture, 0.2 µCi [^3^H] thymidine (Amersham Biosciences, Amersham, UK) was added to each well and the incorporation of radioactivity was measured after 16 hours using liquid scintillation counting.

### MTT assay

OE33, OE19 and SW620 cells were treated with several concentrations of trastuzumab, namely 2.5, 5 and 10 µg/ml, or left untreated for control purposes. Cells were treated for 24 hours or 72 hours, transferred on an enzymatic 96 wells plate and co-incubated with MTT[3-(4,5-dimethylthiazol-2-yl)-2,5-diphenyltetrazolium bromide], for 4 hours. Ethanol absolute (96%) was added to stop the reaction, and results were read on a multi-well scanning spectrophotometer (ELISA reader).

### Apoptosis assay

Each cell line (1×10^4^ cells) was cultured in 2 ml of RPMI 1640 and DMEM as appropriate with or without trastuzumab (10 µg/mL) at 37°C for 48 hours in a six-well plate. After incubation, cells were harvested, spun down, and stained for FACS analysis as described previously [26]. Apoptosis of the trastuzumab treated versus non treated cells was measured by using the following antibodies: anti-human Annexin-V APC conjugated (ICQ, Groningen, The Netherlands), in order to quantify apoptosis; Via-probe 7aad (necrosis marker; R&D System), in order to exclude necrotic cells from the final analysis. Data were acquired using BD Cell Quest Pro Software. Apoptotic epithelial cells were gated as positive for AnnexinV and negative for 7aad and as positive when compared to the isotype control antibody for Annexin V.

### Cytotoxicity assay

The HLA-A2 positive/HER-2 positive OE33 and OE19 cell lines and the HLA-A2 positive/HER-2 negative SW620 cell line were pre-incubated for 48 hours with 10 µg/ml trastuzumab. Hereupon, 1×10^4^ treated and non treated cells (target) were co-incubated with effector cells i.e., the HLA matched HER-2 specific CTLs or non specific CTLs (lymphocytes stimulated with mock transfected DCs), at various E:T ratios, namely 5∶1, 2.5∶1, 1.25∶1 and 0.62∶1, in 100 µl of RPMI 1640 in a 96-well plates in triplicate for 4 hours at 37°C.

The percentage of cytotoxicity was measured by the CytoTox 96 Non-Radioactive Cytotoxicity assay (Promega, Madison, USA). This assay quantitatively measures lactate dehydrogenase (LDH), a cytosolic enzyme that is released upon cell lysis. Released LDH in culture supernatant is measured with a 30-minute coupled enzymatic assay, which results in the conversion of a tetrazolium salt (INT) into a red formazan product. The amount of colour is proportional to the number of lysed cells. Wavelength absorbance data was collected using a multi-well scanning spectrophotometer (ELISA reader). The percentage of specific cytotoxicity was calculated according to the formula: % specific lysis =  [(Experimental-Effector Spontaneous-Target Spontaneous)/(Target Maximum-Target Spontaneous)] ×100.

### Cytometric Bead Array

Supernatants from the different samples were collected after performing the cytotoxicity assays. These were used for measuring IFN-γ release from the activated HER-2 specific CTLs and control lymphocytes in the co-culture/cytotoxicity experiments as described above. The procedure was followed as described previously [30], following the manufacturer's instruction and samples were examined in triplicate for each condition.

### TAP-1, TAP-2 and Tapasin expression in EAC cell lines and EAC patient biopsies

To evaluate the status of three of the most important components of the APM, namely, TAP-1, TAP-2 and Tapasin, RT-PCR, ICC and IHC were performed on OE33, OE19 and SW620 cell lines and on 16 EAC patient biopsies.

RNA from cell lines was isolated by using the Qiagen RNA isolation Kit following manufacturer's instructions (QIAGEN Benelux B.V., The Netherlands) and preserved at −80°C until use. RNA from the EAC patient biopsy specimens was isolated performing the TRiZol Isolation method as described previously [26]. Isolation was performed using the manufacturer's instructions.

Specific primers used for TAP-1, TAP-2 and Tapasin RT-PCR were as follows: TAP-1 fw: AGTGGGAATCCTCTACATTGGTG; rv: TTGGGTAGGCAAAGGAGACA. TAP-2 fw: CTCGTTGCCGCCTTCTTCT; rv: AGTTCAGCTCCCCTGTCTTAGTC. Tapasin fw: CCACCATACACCTGCCATACC; rv: CCCAGTGCCTTGAAGAGCC [31]. The PCR program settings were as follows: 5′95°, 35×(1′95°, 1′58°, 2′72°) 10′72°.

IHC and ICC for the three proteins were performed both on cell lines and EAC patient material as described above, using specific antibodies for TAP-1 (Abcam, Cambridge, UK), TAP-2 (AMS Biotechnology, Milton Park, Milton, UK) and Tapasin (Abcam, Cambridge, UK) at dilutions of 1∶50, 1∶50 and 1∶100 respectively.

### IFN-γ treatment and incubation with IFN- γ producing HER-2 specific CTLs

OE19 cell line was treated for 24, 48 and 72 hours with 5, 10 and 20 ng of IFN-γ (Roche, The Netherlands). Subsequently, RNA from the treated cells was isolated by using the Qiagen RNA isolation kit, as described above, and RT-PCR was performed to evaluate TAP-2 expression before and after IFN-γ treatment. OE19 cells either treated or not with IFN-γ were analyzed by FACS for measuring the expression of HLA-A2.

To study re-expression of TAP-2 under more physiological conditions, the OE19 cell line was also incubated for 48 hours with HER-2 specific IFN-γ producing CTLs. Restoration of TAP-2 expression was monitored by ICC by using a specific antibody for TAP-2 (AMS Biotechnology, Milton Park, Milton, UK). The staining procedure was performed as described above. IFN-γ production by CTLs was measured by performing a CBA assay as described above.

### TAP-2 RNA interference in OE33

The OE33 cell line was seeded at a density of 2.5×10^5^ cells/well in 24 well plates before transfection. Cells were left overnight at 37°C for attachment; subsequently, culture media was replaced with 1 ml antibiotic-free media 2 h before transfection. Cells were placed in a transfection mixture containing 400 µl of antibiotic-free media (Opti-Mem without antibiotics) and 100 µl of transfection mixture, prepared with 8 µl siRNA against TAP-2 (TAP2 siRNA, Santa Cruz Biotechnology, 10 µM) or 3 µl of scrambled RNA diluted in 42 µl Opti-Mem with 0,5% serum (and 47 µl, respectively), mixed with 2 µl DharmaFECT siRNA Transfection Reagent 1 (Dharmacon RNA technology, Perbio Science, Nederland) diluted in 48 µl Opti-Mem. Media was changed after 24 h and replaced with normal culture medium (RPMI 1640) with 10% FCS. After 48 hours, cells were washed with PBS and harvested in Laemmly sample buffer. The lysates were used to load 30 µl of each sample per/lane, resolved by polyacrylamide gel electrophoresis and probed by Western blotting using primary antibodies specific against human TAP-2 (AMS Biotechnology, Milton Park, Milton, UK), and β-actin (Santa Cruz) and a chemiluminescent detection system (Roche, The Netherlands). The cells, which displayed down-regulation of TAP-2, where subsequently used in the cytotoxicity assay (see experimental procedure above). The down-regulation of TAP-2 in OE33 was also analyzed by ICC (procedure see above).

### Statistics

To evaluate statistical differences between groups, Student's *t* test and Anova followed by Bonferroni post test for multiple comparisons were used. Statistically significant difference was considered at p<0.05.

## Results

### HER-2 expression and gene amplification in EAC cell lines

**Figure 1:**
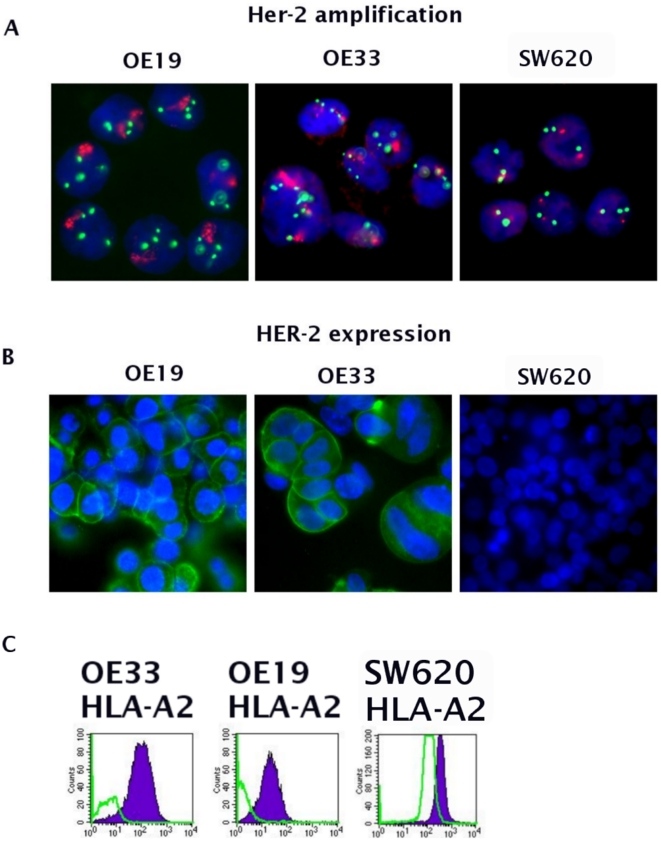
HER-2 status in EAC cell lines. HER-2 status in the EAC cell lines shows that OE33 and OE19 are characterized by high HER-2 gene amplification, whereas SW620 exhibits polysomy of chromosome 17 only (A). On protein level, OE33 and OE19 both overexpress HER-2. The SW620 cell line does not over-express HER-2 (B). HLA-A2 status in OE33, OE19 and SW620 cell lines (violet = HLA-A2 expression; green = isotype control, C).

Determination of the HER-2 status in the OE33, OE19 EAC cell lines and SW620 by DNA FISH revealed that OE33 and OE19 are characterized by a high HER-2 amplification as compared to SW620 that only showed polysomy of chromosome 17 (Fig.1A, Table 1). This was confirmed on the protein levels by ICC, which showed a strong overexpression of HER-2 in OE33, a weaker expression in OE19, whereas SW620 (control) showed no staining (Fig.1B). The results on HER-2 expression observed by ICC were further confirmed by FACS analysis (data not shown). The HLA-A2 status of the three cell lines used was determined by FACS analysis and resulted to be positive in all the three cell lines (Fig. 1C, supplementary fig. S3).

**Table 1:** EAC Patients information.

Patients	Age	Sex	Type of cancer	Classification
1	51	male	EAC	G2T3N1M1b
2	75	male	EAC	T3N0
3	50	male	EAC	T1N0
4	71	male	EAC	T3N1
5	53	male	EAC	T1N0M0
6	73	male	EAC	T3N0M0
7	80	male	EAC	T3N0M0
8	76	female	EAC	T1N0
9	58	male	EAC	T1N0
10	68	male	EAC	T4N1
11	76	female	EAC	T1N0
12	60	male	EAC	T1N0M0
13	82	male	EAC	T3N1M0
14	85	male	EAC	T3N1
15	78	male	EAC	T3/4N1
16	80	male	EAC	T1N0
17	77	male	EAC	T3N1M0

### Trastuzumab inhibits growth, but does not increase apoptosis, in HER-2 overexpressing EAC cell lines

**Figure 2:**
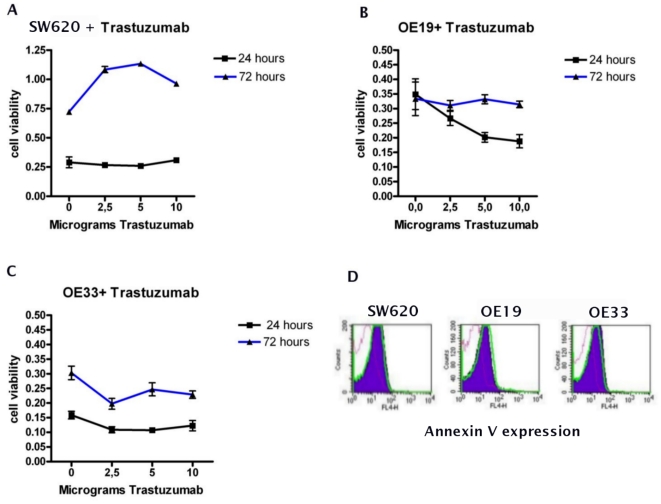
The biological activity of trastuzumab on EAC cell lines. The HER-2 positive EAC cell lines treated with increasing concentrations of trastuzumab for 24 and 72 hours, progressively show decreased cell viability in both OE33 and OE19 (A and B). SW620 (HER-2 negative colon cell line) shows no sensitivity to trastuzumab (C) (Data are means ± SD of six experimental points from two independent experiments). Apoptosis levels as measured in EAC cell lines treated for 48 hours with 10 µg of trastuzumab does not show any difference between treated and not treated cells (Violet area D =  trastuzumab treated cells, Green line D =  not treated cells, dotted line D =  isotype control).

To evaluate the potential inhibitory activity of trastuzumab on the HER-2 overexpressing cell lines, OE33, OE19 and the non HER-2 overexpressing SW620 were treated with different concentrations of trastuzumab for 24 and 72 hours. Hereafter, MTT assays were performed to measure the growth inhibitory effect of trastuzumab and cells were stained for Annexin V and 7aad to assess the level of apoptosis. Cells, which were not treated with trastuzumab, showed a basal cell viability of on average 0.21±0.02SEM for OE33 and on average 0.34±0.02SEM for OE19. Incubations for 24 and 72 hours with trastuzumab already showed significant reductions in cell growth in OE33 and OE19 at a dosage of 2.5 µg of trastuzumab (Paired T-test, p<0.05). Increasing the dosage however, did not further affect cell viability in OE33, but did decrease cell growth in OE19 (Fig.2A, B). In SW620, a HER-2 negative colon cell line, no differences were observed in cell viability for any incubation time using different concentrations of trastuzumab (Fig.2C).

Annexin V and 7aad stainings performed to assess the apoptosis level induced in trastuzumab treated EAC cell lines, showed no significant increase in apoptosis as compared to non-treated cells (Fig.2D). Violet areas indicate the population of treated cells, green lines indicate the population of non treated cells, and purple dotted lines indicate the population stained with the isotype control for the Annexin V antibody.

### Mixed Leukocyte Reaction (MLR) with HER-2 specific CTLs

**Figure 3:**
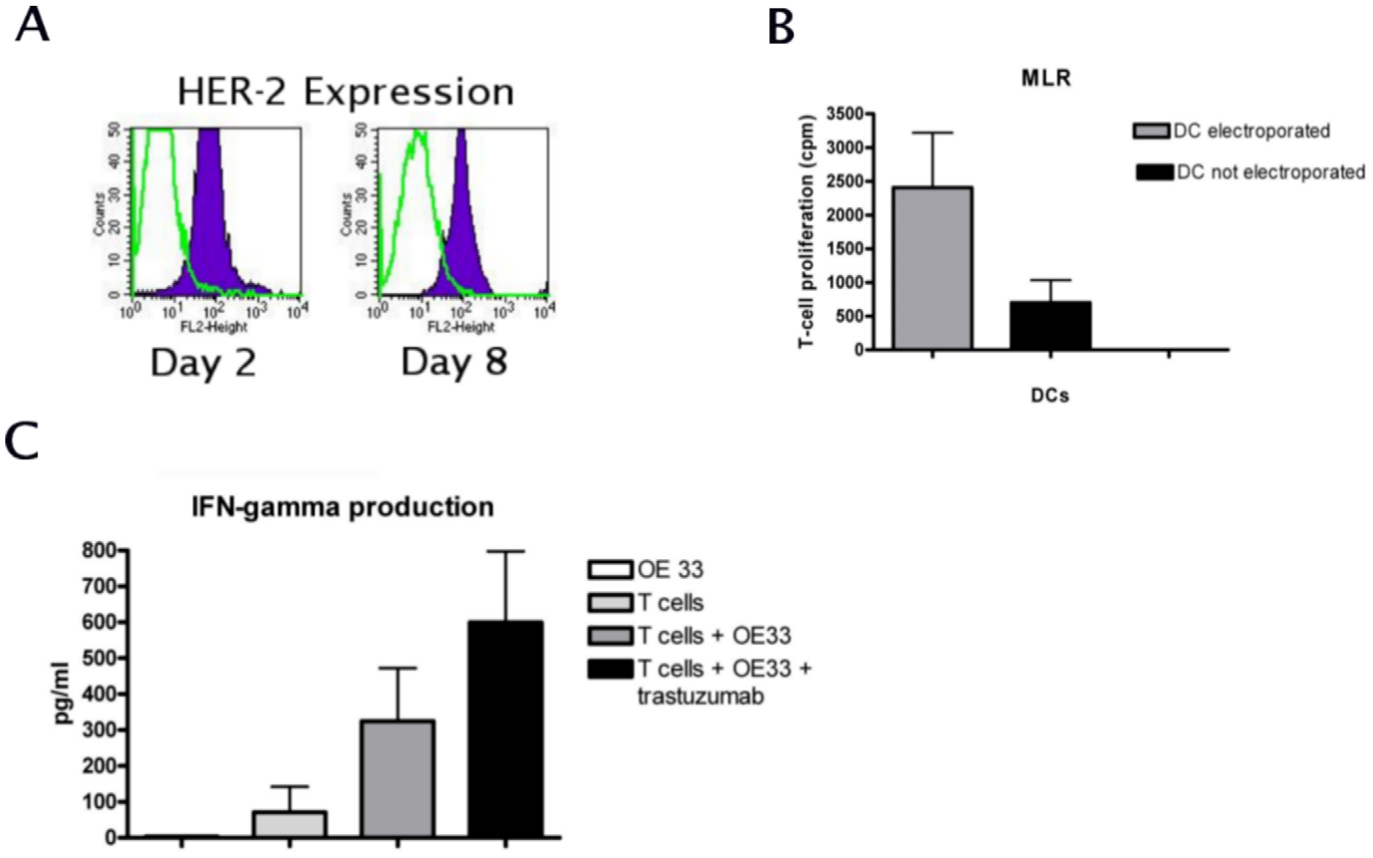
Detection of HER-2 epitopes and HER-2 specific CTLs. Monocytes from HLA−A2+ donors were electroporated with IVT HER-2 RNA. FACS analysis shows that after 24 hours and 8 days, HER-2 epitopes were specifically expressed in electroporated cells, while cells stained with an isotype control and secondary antibody alone were virtually negative (Paired T-test, p<0.05, A, figures are representative of three independent experiments). MLR of lymphocytes with either HER-2 electroporated DCs, or DCs, which underwent the electroporation (mock) procedure without HER-2, shows that HER-2 electroporated DCs were able to induce significantly higher T-cell proliferation, as compared to the mock electroporated DCs (Paired T-test, p<0.05, B, n = 2). IFN-γ release by CTLs incubated with HER-2 overexpressing OE33 cell line was significantly higher as compared to CTLs alone (T-test, p<0.05). The release increased in case OE33 was pre-treated with trastuzumab (T-test, p<0.05, C, n = 3).

FACS analysis performed using the HER-2 specific antibody, confirmed that 24 hours after electroporation, HER-2 was successfully expressed by the HER-2 RNA loaded DCs. At day eight of the maturation process the mature DCs were still able to present HER-2 antigens, as demonstrated by a significantly higher MFI of 1608±448SEM as compared to the control isotype antibody and mock electroporated cells (MFI = 167±70 and 112±38, respectively, Paired T-test, p<0.01, Fig. 3A). In the MLR, lymphocytes, which were stimulated by HER-2 loaded DCs showed a significantly higher proliferation rate (Mean 2409±809SEM) as compared to the lymphocytes stimulated by mock-electroporated DCs (Mean 702±131SEM, Paired T-test, p<0.05, Fig.3B). The resulting anti HER-2 specific CTL population and control CTL population (lymphocytes stimulated with mock transfected DC) were subsequently used for the cytotoxicity assays.

### IFN-γ release by CTLs after cytotoxicity assay

After four hours of incubation for the cytotoxicity assay, the supernatant obtained from the co-cultures of CTLs and the EAC cell line OE33 either treated or not treated with trastuzumab was collected and used to measure the IFN-γ release by the CTLs performing CBA. Production of IFN-γ was significantly higher when CTLs were co-incubated with OE33 without trastuzumab pre-treatment, 324.8 pg/ml ±147.5SEM, as compared to the basal IFN- γ levels, 71.17 pg/ml±71.7SEM, T-test, p<0.05 (Fig. 3C). In case OE33 cells were pre-treated with trastuzumab, the production of IFN-γ from the CTLs was significantly higher as compared to the CTLs' basal level of IFN-γ production, 599.6 pg/ml±197.9SEM (T-test, p<0.05). These results correlated with the lytic activity as observed in the cytotoxicity assays.

### Trastuzumab sensitizes OE33 but not OE19 to HER-2 specific CTL lysis

**Figure 4:**
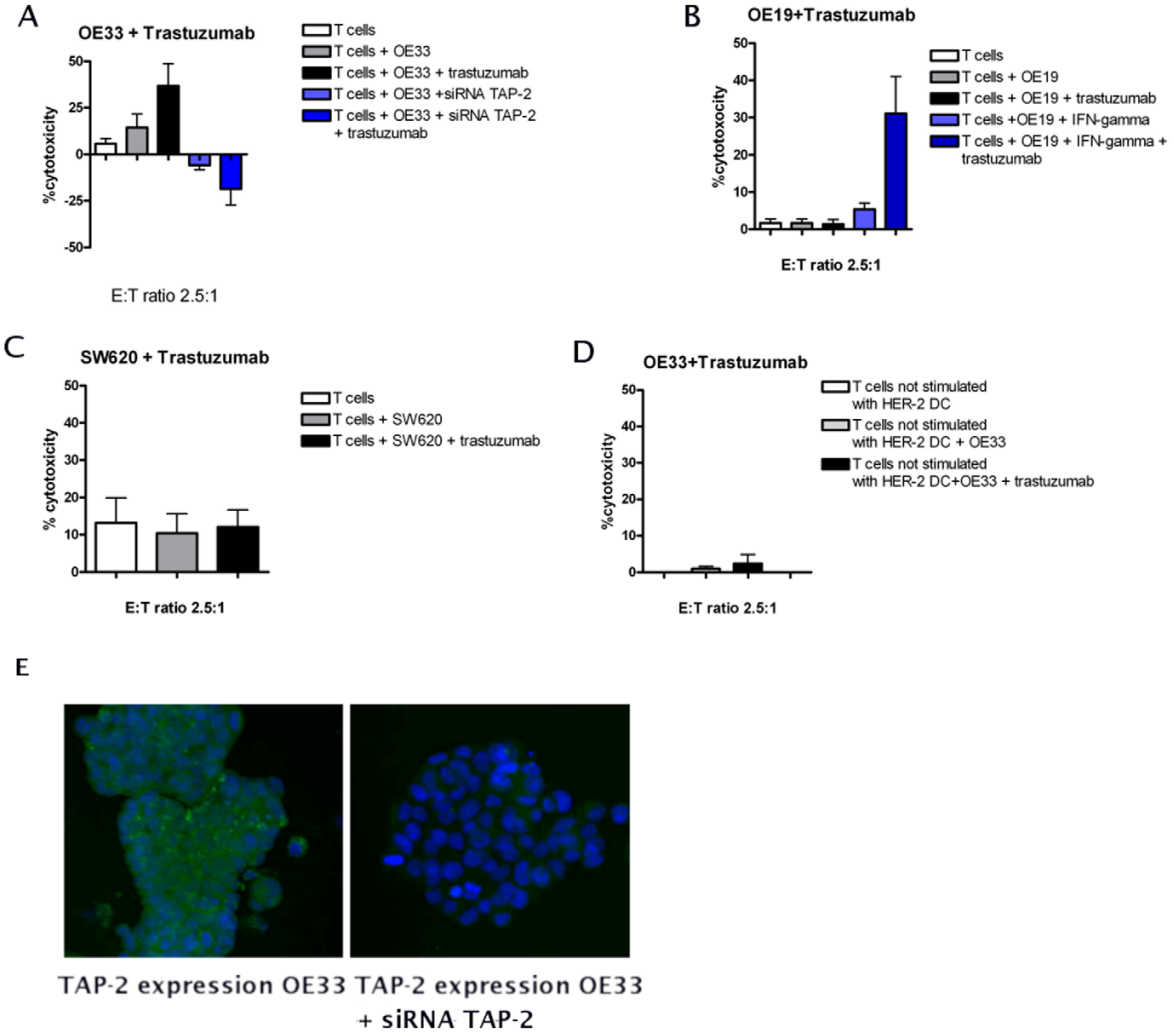
The sensitizing effect of trastuzumab. The cytotoxic capacity of HER-2 specific CTL populations was tested on the HER-2 positive EAC cell lines pre-treated with trastuzumab. Results show that trastuzumab pre-treatment significantly enhances the sensitivity of OE33 cells to the HER-2 CTL lysis activity (A). This effect is not observed in OE19 cells, despite that this cell line does express HER-2 (B). SW620, which is HLA-A2 positive, but HER-2 negative as expected does not show any sensitivity to trastuzumab treatment (C). Data are means ± SEM of three experimental points from six independent experiments. Simultaneous treatment of OE19 with IFN-γ and trastuzumab restored the cytotoxicity effect induced by HER-2 specific CTLs (C). Incubating OE33 cells with non-specific T cell population did not show any enhanced cytotoxic effect (D). SiRNA mediated inhibition of TAP-2 expression in OE33 was successfully obtained as seen by ICC (E). Silencing of TAP-2 in OE33 with siRNA results in a significantly decreased CTL response of these cells independent of pre-treatment with trastuzumab as compared to OE33 which were not transfected with siRNA for TAP-2 (A).

The cytolytic effects of different CTL populations on trastuzumab treated and non treated EAC cell lines were studied. The overall percentage of lysis induced by the HER-2 specific CTLs on the HER-2 overexpressing EAC cell line OE33 was 14.4%±7.1SEM, while the effect obtained with (control) aspecific CTLs was 1%±0.7SEM. After pre-treatment of the cell lines with trastuzumab the lysis of the OE33 cell line increased to 36,8%±11.8SEM, while the effect induced by aspecific CTLs was 2.4±2.4SEM (Fig.4A and D, n = 6). Under these different conditions a mean cytotoxicity level of 2.2±1.1SEM was measured for the OE19 cells (Fig.4B). Thus there was hardly any cytolytic effect induced by the HER-2 specific CTLs or any sensitizing effect of the trastuzumab pre-treatment observed on the HER-2 over-expressing OE19 cells. As expected, SW620, which does not over-express HER-2, did not show any sensitivity to the anti HER-2 specific CTLs with or without trastuzumab pre-treatment (mean cytotoxicity in all the samples = 12±1.5SEM, Fig.4C). In the control experiments, lymphocytes stimulated by mock-transfected DCs, did not exhibit a cytotoxic effect on OE33, either treated or not with trastuzumab (Fig. 4D).

### IFN-γ restores the HER-2 specific CTL lysis of OE19

Upon IFN-γ treatment, the cytotoxic capacity of the HER-2 specific CTLs towards OE19 cell line was restored and increased from 1.6±1.05 to 5.3±1.7SEM (Fig. 4C, one way Anova, Bonferroni post test, p = 0,003). With IFN-γ treatment the sensitizing effect of trastuzumab was as well restored. Simultaneous treatment with IFN-γ and trastuzumab further increased the mean cytotoxicity to 31.1±9.9SEM (Fig. 4B, One way Anova, Bonferroni post test, p<0,05).

### Inhibition of TAP-2 expression in OE33 reduces the synergistic cytotoxic effect of trastuzumab and HER-2 specific CTLs

In order to further confirm that the synergistic cytotoxic effect induced by simultaneous treatment of trastuzumab and HER-2 specific CTLs is dependent on TAP-2, we inhibited the expression of TAP-2 in OE33 cell line by siRNA. We subsequently performed a cytotoxicity assay on these cells, with or without Trastuzumab pre-treatment. First we showed by ICC and Western blot that inhibition of TAP-2 expression was successfully obtained on the TAP-2 siRNA treated cells (Fig. 4E and supplementary fig. S6). By incubating these TAP-2 deficient cells with HER-2 specific CTLs and trastuzumab, we could observe that the CTL induced cytotoxicity in both trastuzumab treated and not treated cells is decreased as compared to cells in which the TAP-2 was not inhibited, and the % of lysis was on average −5,8±2,6 for the untreated cells, and −18,6±8,7 (n = 2) (Fig. 4A).

### TAP-1, 2 and Tapasin expression in EAC cell lines and EAC patient material

**Figure 5:**
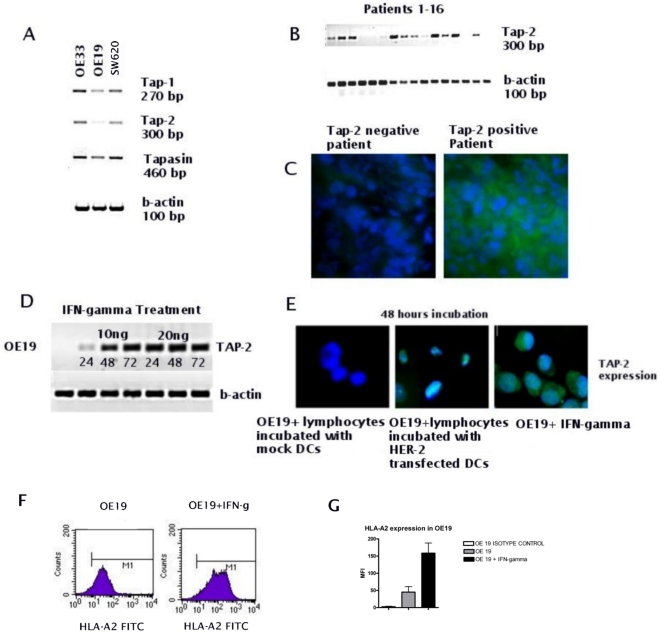
APM status in EAC cell lines and patient biopsies and re-induction of TAP-2 by IFN-γ and INF-γ producing CTLs. TAP-1, TAP-2 and Tapasin expression at RNA and protein level were detected by RT-PCR and IHC/ICC, respectively. TAP-1 and Tapasin are normally expressed in OE19, while expression of TAP-2 is absent (A). RT-PCR shows that TAP-2 expression is lost or down-regulated in 70% of the tumor biopsies of EAC patients (B). IHC on EAC biopsies of the same patients supports the RT-PCR results (C). RT-PCR shows re-induction of TAP-2 in OE19 after time course incubations with 5, 10 or 20 ng of IFN-γ (D). Co-incubation of OE19 with INF-γ producing CTLs for 48 hours also restores TAP-2 expression (E). OE19 cells treated with IFN- γ displays a significantly increased HLA-A2 expression as compared to not treated cells (MFI = 45±16 versus 158±29, F and G).

To investigate the reason accounting for the deficient cytotoxic response observed on OE19, expression of three important components of the APM, TAP-1, TAP-2 and Tapasin, was determined by RT-PCR, ICC and IHC in the EAC cell lines and EAC patient material. In all three cell lines at RNA level, there was normal expression of TAP-1 and Tapasin, while TAP-2 expression was absent in OE19 but positive in OE33 and SW620 (Fig.5A). ICC supported the RT-PCR results (see supplementary fig. S7) and showed low expression of the TAP-2 protein in OE19 (see Fig. 5E). To look at the status of TAP-2 in patient material, RT-PCR was performed on RNA isolated from 16 EAC biopsies, and IHC was performed on correlating biopsies of the 16 EAC patients. In 70% of the patients there was either a complete or a partial down-regulation of TAP-2 expression at both RNA and protein level (Fig. 5B and C). RT-PCR and IHC was performed on EAC biopsies to check as well the expression of TAP-1 and Tapasin. In 70% of the biopsies specimens we found down-regulation and/or complete lack of TAP-1 expression, and in 40% down-regulation and/or lack of expression of Tapasin (see supplementary fig. S1 and S2).

### IFN-γ and IFN-γ producing CTLs restore TAP-2 expression in OE19

To establish whether the TAP-2 deficiency displayed by OE19 is due to a transient down-regulation, OE19 was treated at several time points with different concentrations of IFN-γ: after 24 hours it was already possible to observe an up-regulation of TAP-2 in OE19, which notably increased after 48 hours (Fig. 5D).

Since the anti HER-2 CTL populations as raised by HER-2 RNA loaded DCs have the potential to produce high levels of INF-γ (see supplementary fig. S5), we incubated OE19 cells with these specific CTLs for 48 hours. After a prolonged incubation of 24 hours with the IFN-γ producing HER-2 specific CTLs, we were able to observe a restored TAP-2 expression, as shown by ICC analysis (Fig. 5E). FACS analysis performed on OE19 cells either treated or not with IFN-γ, showed that the HLA-A2 expression increased significantly in the cells which were treated with IFN-γ (MFI = 45±16 versus 158±29, Fig. 5F and G and supplementary fig. S3, n = 2).

## Discussion

The present report contains several important findings regarding the underlying mechanism of action of the antibody trastuzumab on HER-2 overexpressing EAC. Moreover, it proves that an intact Antigen Processing Machinery (APM) in these cancer cells is of pivotal importance for an optimal effect of trastuzumab and for T-cell mediated immunotherapies. It is well known that deficiencies in the expression of APM components can hinder or entirely abolish anticancer immune-responses by preventing proper CTL-induced cell lysis as a consequence of a reduced level of MHC-Class I antigen presentation [32], [33], [34]. In this regard, the sensitization to tumor cell lysis by MHC-Class I restricted CTLs promoted by trastuzumab is dependent on a proper APM function. For the above mentioned effect, it has previously been shown that trastuzumab, by binding the HER-2 receptor, increases internalization and degradation of the latter, which might augment the amount of HER-2-derived peptides available for loading to MHC class I [24], [25]. Previously, Kono et al, performed several studies to determine the exact mechanism of trastuzumab, and they demonstrated that DCs pulsed with HER-2/neu-derived peptides can induce specific T-cell responses in patients with gastric cancer when these are pre-treated with trastuzumab [12]. The same group demonstrated that trastuzumab is able to induce a cytolytic response against HER-2–expressing esophageal Squamous Cell Carcinoma (SCC) [35]. In addition, recent studies demonstrated that HER-2 specific CTLs, generated from HLA-A2 positive patients with HER-2 positive breast cancer, can lyse HER-2 positive SKOV3tA2 breast cancer cells in a HLA-A2 restricted manner [25]. These results suggest that HER-2 specific CTLs can induce lysis of trastuzumab sensitized HER-2-overexpressing tumors in a MHC-Class I restricted manner [24].

In our study, we examined the HER-2 status of several EAC cell lines and showed that OE33 and OE19 are characterized by high levels of HER-2 gene amplification, confirming previous findings [9], whereas SW620, a HLA-A2 positive colon cancer cell line taken as control, is characterized by only polysomy of chromosome 17. These results correlated with the protein expression levels as seen by ICC. As expected trastuzumab significantly reduce cell viability and proliferation in OE33 and OE19 as compared to not treated samples, whereas SW620 is not affected by trastuzumab. In contrast, we found that trastuzumab treatment does not result in an increase in apoptosis of OE33 and OE19, confirming prior observations [24]. To find out whether trastuzumab is able to induce sensitization of the HER-2 positive EAC cell lines to HER-2 specific CTLs induced lysis, we co-cultured HER-2 specific CTLs with EAC cells treated or not with trastuzumab, and we found that treatment with trastuzumab significantly sensitized the HER-2 overexpressing EAC cell line OE33 to HER-2 specific MHC-Class I restricted CTL lysis. Yet, to our surprise, this effect was not observed for the other HER-2 overexpressing cell line OE19. Since CTL mediated cytotoxicity is dependent on presentation of antigens through an intact APM, we investigated the status of the APM in the EAC cell lines and EAC patient biopsy specimens. Amongst the APM components, the Transporter associated with Antigen Processing 1 and 2 (TAP-1 and 2) polymorphisms and mutations are associated with hindered anticancer responses and poor patient prognosis [36]. TAP-1 and TAP-2 down-regulation in general will lead to a reduced efficiency in antigen presentation and immune-responses [37] and is considered as an important mechanism of cancer immune-escape [38]. We found normal expression levels of TAP-1 and Tapasin but significant down-regulation of TAP-2 in OE19. Moreover, absent or decreased expression of TAP-2 protein was seen in 70% of the EAC cases. It is well known that components of the APM feature an IFN-γ response element on their genes, therefore IFN-γ is the most potent up-regulator of TAP-1 and TAP-2 expression in case these protein are de-regulated, and not mutated [39]. By RT-PCR and ICC we showed that IFN-γ and co-incubation of OE19 with IFN-γ producing anti-HER-2 CTLs efficiently restored TAP-2 expression. Importantly, by performing FACS analysis we found that expression of MHC-Class I molecules on OE19 is increased upon treatment with IFN-γ. Next we also showed that the HER-2 specific CTL induced cytotoxicity towards OE19 was restored after treatment with IFN-γ. Moreover, we observed that simultaneous INF-γ and trastuzumab treatment resulted in significantly increased cell lysis of OE19 as compared to the IFN-γ treatment alone. Conversely, we demonstrated that this mechanism of action of trastuzumab is dependent on a proper TAP-2 expression, since down-regulation of this APM component by siRNA in OE33, resulted in an expected reduced CTL cytotoxicity, but also abolished the synergistic effect of trastuzumab and T-cells. Our observation that TAP-2 down-regulation is frequently seen in EAC patients indicates that in a clinical setting an impaired APM function could hamper patient response to immunotherapy and the sensitizing effect of trastuzumab. An important direction to solve this important issue would be to combine trastuzumab with DC immunotherapy or IFN-γ. DCs may induce anti-HER-2 CTLs with the capacity of producing high INF-γ levels. Once these CTLs invade the tumor environment and target the tumor cells, INF-γ release could re-establish TAP-2 expression. In turn, trastuzumab would highly enhance antigen presentation, which would sensitize the cells and target them to CTL-induced tumor cell lysis. Finally, it is important to note that NK cells might also be involved in the response of the patient to trastuzumab as treatment of OE33 with trastuzumab increases HER-2 positive PBMCs induced antibody-dependent cell-mediated cytotoxicity (ADCC) on OE33 as compared to untreated cells. Conversely, this phenomenon is not observed when OE33 are incubated with PBMCs isolated from an HER-2 negative patient (supplementary fig. S4). These observations confirm previous results obtained in gastric carcinoma and esophageal squamous carcinoma from the group of Kono et al. [13], [22] where it was shown that trastuzumab act as an inducer of the ADCC mechanism.

We conclude that a proper function of the APM is necessary for both an effective CTL response and for inducing an optimal immune response to trastuzumab. Of importance is the fact that potent IFN-γ producing CTLs by themselves may reinstate down regulated TAP and facilitate cytotoxicity. In the future it would be highly interesting to evaluate the APM status of patients who are treated with trastuzumab or T cell-mediated immunotherapies and to investigate whether this truly correlates with tumor responsiveness and long term patient outcomes. Our data offers convincing arguments for treating EAC patients carrying HER-2 overexpression with a combinatorial therapy consisting of trastuzumab and IFN-γ or anti-HER-2 directed T cell-mediated immunotherapy.

## Supporting Information

Figure S1 To evaluate the status of three of the most important components of the APM, namely, TAP-1, TAP-2 and Tapasin, RT-PCR, ICC and IHC were performed on OE33, OE19 and SW620 cell lines and on 16 EAC patient biopsies. IHC for the three proteins were performed EAC patient material using specific antibodies for TAP-1, TAP-2 and Tapasin at dilutions of 1∶50, 1∶50 and 1∶100 respectively. In this figures there are representative examples of patients which resulted negative or positive to the TAP-1 and the Tapasin staining.
(1.08 MB TIF)

Figure S2 To evaluate the status of three of the most important components of the APM, namely, TAP-1, TAP-2 and Tapasin, RT-PCR was performed on 16 EAC patient biopsies. 70% of the patients showed lack and/or down-regulation of the expression of both TAP-1 and/or TAP-2, and a minority (6 out of 16) showed lack or down-regulation of the expression of Tapasin.
(0.54 MB TIF)

Figure S3 HLA-A2 status was checked by performing FACS analysis of OE33 and OE19. Although both OE19 and OE33 resulted to be HLA-A2 positive, OE19 showed a significantly lower expression of HLA-A2. After treatment with IFN-γ, the level of expression of HLA-A2 in OE19 significantly increased.
(0.19 MB TIF)

Figure S4 PBMCs isolated either from HER-2 positive patients and HER-2 negative patients were incubated with OE33 treated or not treated with Trastuzumab and subsequently a cytotoxicity assay was performed. It is possible to observe that PBMCs isolated from HER-2 positive patients induced a significantly higher cytotoxicity, probably due to antibody-dependent cell-mediated cytotoxicity (ADCC) on OE33 as compared to untreated cells. Conversely, this phenomenon is not observed when OE33 are incubated with PBMCs isolated from an HER-2 negative patient.
(1.69 MB TIF)

Figure S5 IFN-γ production from the CTLs which were co-incubated with OE19 in order to up-regulate TAP-2 expression was checked by CBA and a significantly higher production was detected from the CTLs incubated with HER-2 RNA transfected DC as compared to T cells incubated with mock transfected DC.
(0.75 MB TIF)

Figure S6 In order to inhibit TAP-2 expression, OE33 cell line was transfected with a siRNA mixture targeting TAP-2. By western blot and ICC we showed that TAP-2 expression was successfully down-regulated in the cells which were transfected with siRNA for TAP-2 as compared to those which were not transfected.
(0.07 MB TIF)

Figure S7 To evaluate the status of three of the most important components of the APM, namely, TAP-1, TAP-2 and Tapasin, ICC was performed on OE33 and OE19. In both the cells lines on protein level there was normal expression of TAP-1 and Tapasin, whereas TAP-2 expression was absent in OE19. These results confirmed the RT-PCR data.
(3.00 MB TIF)
