# Supplementary figures and images for: Trastuzumab Mediated T-Cell Response against HER-2/Neu Overexpressing Esophageal Adenocarcinoma Depends on Intact Antigen Processing Machinery
Source: PLoS One. 2010 Aug 26;5(8):e12424. doi: 10.1371/journal.pone.0012424 (PMC2928738; doi:10.1371/journal.pone.0012424)

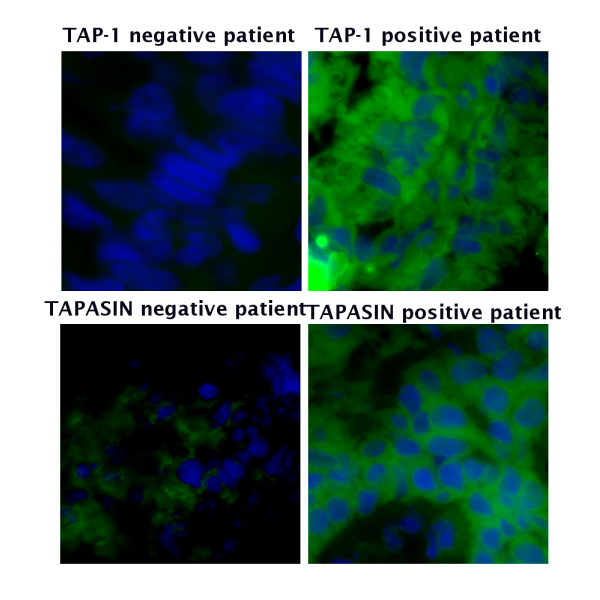

Supplement: Figure S1 — To evaluate the status of three of the most important components of the APM, namely, TAP-1, TAP-2 and Tapasin, RT-PCR, ICC and IHC were performed on OE33, OE19 and SW620 cell lines and on 16 EAC patient biopsies. IHC for the three proteins were performed EAC patient material using specific antibodies for TAP-1, TAP-2 and Tapasin at dilutions of 1∶50, 1∶50 and 1∶100 respectively. In this figures there are representative examples of patients which resulted negative or positive to the TAP-1 and the Tapasin staining. (1.08 MB TIF) [file pone.0012424.s001.tif]

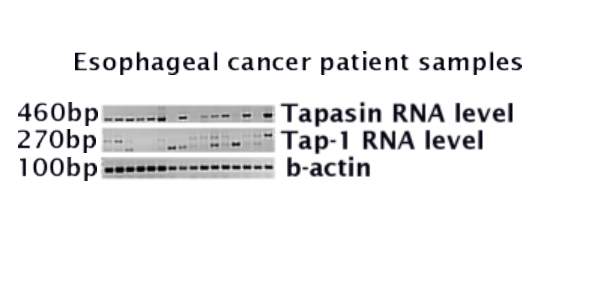

Supplement: Figure S2 — To evaluate the status of three of the most important components of the APM, namely, TAP-1, TAP-2 and Tapasin, RT-PCR was performed on 16 EAC patient biopsies. 70% of the patients showed lack and/or down-regulation of the expression of both TAP-1 and/or TAP-2, and a minority (6 out of 16) showed lack or down-regulation of the expression of Tapasin. (0.54 MB TIF) [file pone.0012424.s002.tif]

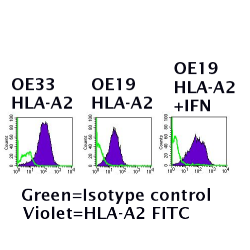

Supplement: Figure S3 — HLA-A2 status was checked by performing FACS analysis of OE33 and OE19. Although both OE19 and OE33 resulted to be HLA-A2 positive, OE19 showed a significantly lower expression of HLA-A2. After treatment with IFN-γ, the level of expression of HLA-A2 in OE19 significantly increased. (0.19 MB TIF) [file pone.0012424.s003.tif]

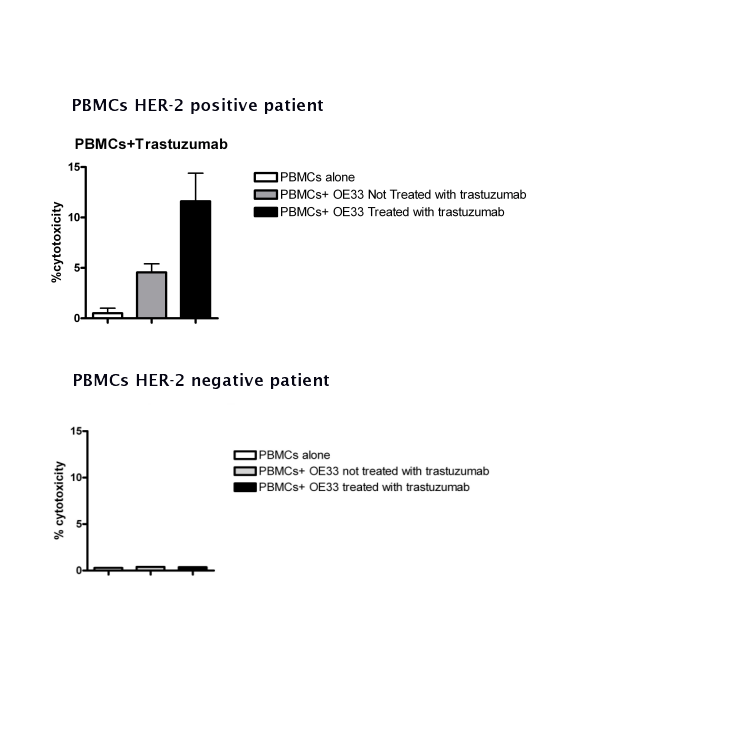

Supplement: Figure S4 — PBMCs isolated either from HER-2 positive patients and HER-2 negative patients were incubated with OE33 treated or not treated with Trastuzumab and subsequently a cytotoxicity assay was performed. It is possible to observe that PBMCs isolated from HER-2 positive patients induced a significantly higher cytotoxicity, probably due to antibody-dependent cell-mediated cytotoxicity (ADCC) on OE33 as compared to untreated cells. Conversely, this phenomenon is not observed when OE33 are incubated with PBMCs isolated from an HER-2 negative patient. (1.69 MB TIF) [file pone.0012424.s004.tif]

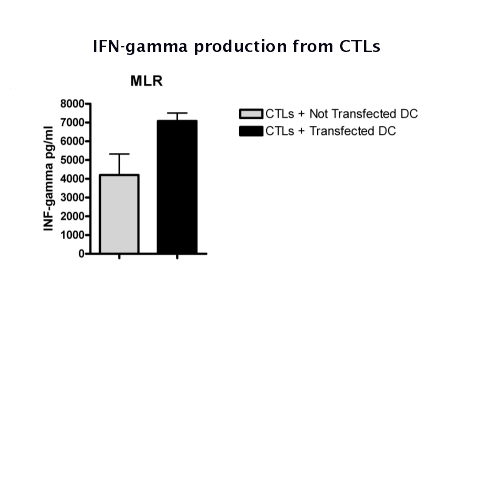

Supplement: Figure S5 — IFN-γ production from the CTLs which were co-incubated with OE19 in order to up-regulate TAP-2 expression was checked by CBA and a significantly higher production was detected from the CTLs incubated with HER-2 RNA transfected DC as compared to T cells incubated with mock transfected DC. (0.75 MB TIF) [file pone.0012424.s005.tif]

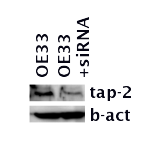

Supplement: Figure S6 — In order to inhibit TAP-2 expression, OE33 cell line was transfected with a siRNA mixture targeting TAP-2. By western blot and ICC we showed that TAP-2 expression was successfully down-regulated in the cells which were transfected with siRNA for TAP-2 as compared to those which were not transfected. (0.07 MB TIF) [file pone.0012424.s006.tif]

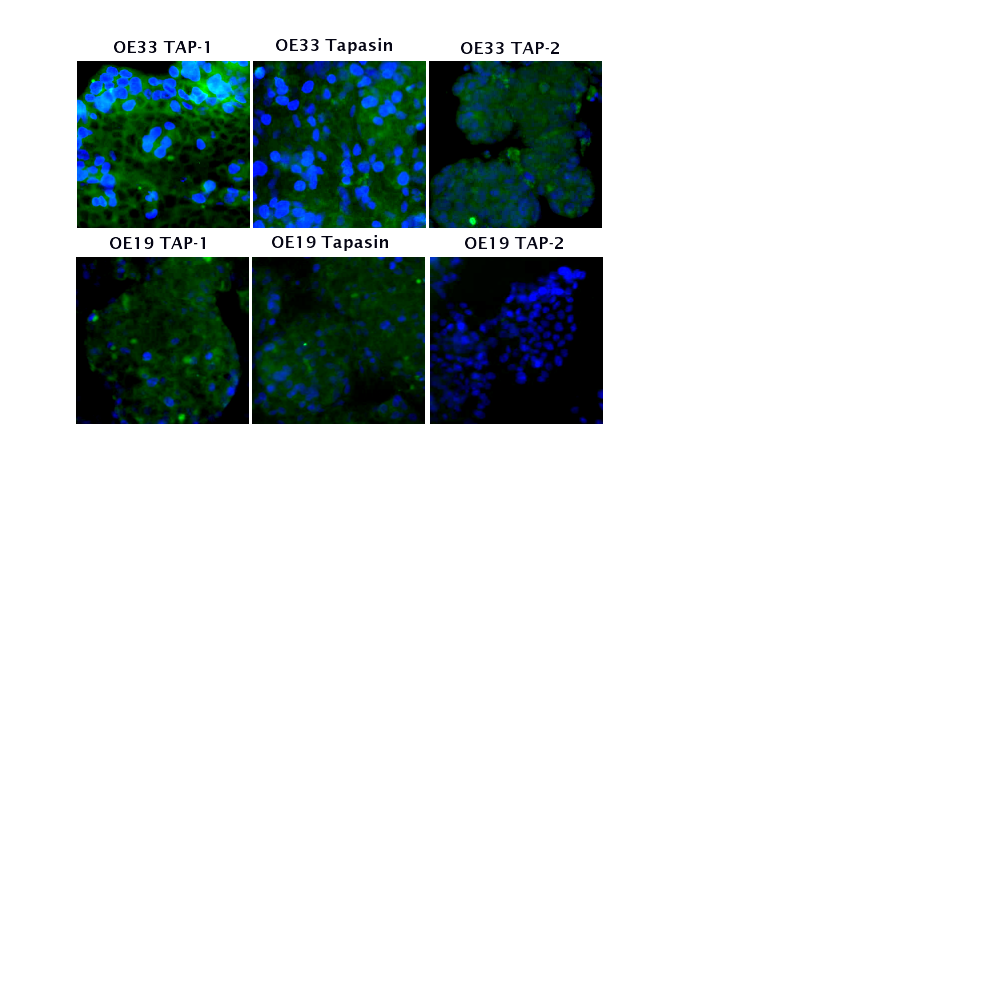

Supplement: Figure S7 — To evaluate the status of three of the most important components of the APM, namely, TAP-1, TAP-2 and Tapasin, ICC was performed on OE33 and OE19. In both the cells lines on protein level there was normal expression of TAP-1 and Tapasin, whereas TAP-2 expression was absent in OE19. These results confirmed the RT-PCR data. (3.00 MB TIF) [file pone.0012424.s007.tif]
